# Assessing the Validity and Reliability of a Questionnaire on Dietary Fibre-related Knowledge in a Turkish Student Population

**DOI:** 10.3329/jhpn.v31i4.20048

**Published:** 2013-12

**Authors:** Melike S. Deniz, Ayten A. Alsaffar

**Affiliations:** ^1^Department of Nutrition and Dietetics, Yeditepe University; ^2^School of Applied Sciences, Ozyegin University, Turkey

**Keywords:** Dietary fibre, Questionnaire, Reliability, Validity, Turkey

## Abstract

This study aimed to validate a questionnaire on dietary fibre (DF)-related knowledge in a Turkish student population. Participants (n=360) were either undergraduate students who have taken a nutrition course for 14 weeks (n=174) or those in another group who have not taken such a nutrition course (n=186). Test-retest reliability, internal reliability, and construct validity of the questionnaire were determined. Overall internal reliability (Cronbach's alpha=0.90) and test-retest reliability (0.90) were high. Significant differences (p<0.001) between the scores of the two groups of students indicated that the questionnaire had satisfactory construct validity. It was found that one-fifth of the students were unsure of the correct answer for any item, and 52.5% of them were not aware that DF had to be consumed on a daily basis. Only 36.4 to 44.2% of the students were able to correctly identify the food sources of DF.

## INTRODUCTION

Dietary fibre (DF) is deemed to be a key component in a healthful diet (1). DF consumption may be important in the prevention or control of cancer, diabetes mellitus, hypertension, hyperlipidaemia, and other health problems (2). There are no accurate data on the daily intake of DF in Turkey. Various studies have reported that the intake of DF by adolescents (3) and university students (4-6) fell below the daily recommended level. The fruit and vegetable consumption by Turkish adults was also found to be inadequate (1.6 portions of fruit and 1.6 portions of vegetables per day) (7).

Consumers need to enhance their knowledge and competence as informed consumers are able to perform their food choices in a complex environment with a wide variety of foods available. They need to be aware of not only the nutritional recommendations and basic food-based guidelines but also how to apply this knowledge in their food choices, diet, and eating behaviour (8). Without adequate knowledge on nutrition, consumers will not be able to make informed choices for their diet and health (2).

Assessing knowledge on nutrition can lead to the identification of deficient areas of information and, thus, would aid in devising nutrition education programmes. Although there are many studies that have measured the general knowledge on nutrition among Turkish people (9-13), we have come across only one study that specifically aimed to measure the knowledge of DF (in addition to other nutrients) among Turkish adults (14). In that study, more than 50% of the participants (n=200) correctly answered four out of five questions that enquired about the dietary sources of DF.

This study aims to describe the development and validation of a new scale that assesses knowledge on DF in a sample of Turkish students. Provided that it has an acceptable reliability and validity, the questionnaire can be used in exploring the level of knowledge on DF among sample of the Turkish population.

## MATERIALS AND METHODS

### Participants

Participants in this study were second-year undergraduate students at Yeditepe University in Istanbul, Turkey. The population in this study included two groups of students: one group had taken a general nutrition course for 14 weeks as a requirement of their curriculum (n=180), and the other group was composed of engineering students (studying computer, electrical-electronics, civil and mechanical engineering) who had not taken such a nutrition course or any other courses relevant to nutrition (n=212). Of them, 174 students who took nutrition courses and 186 engineering students who did not take such courses (total of 360 students) gave written informed consent to take part in the study. The general nutrition course included the function, structure, and sources of macro- and micronutrients, and general principles of healthy eating. Engineering students did not have any specialization in knowledge on nutrition. This ensured that one group had greater knowledge on nutrition.

### Settings

The questionnaire was developed in 2012 in Istanbul, Turkey. Istanbul is one of the largest cities in the world (with a population of approximately 17 million), and it serves as Turkey's economical, social and cultural centre. The university that the study was carried out in is a foundation university and ranks among the most prestigious universities in Turkey.

### Ethical approval

The Research Evaluation Committee of the Department of Nutrition and Dietetics, Yeditepe University, approved the study.

### Questionnaire development and testing

On the basis of review of the current material containing dietary advice and the literature linking DF and health, it was decided to divide the questionnaire into two sections. The first section (Section A) included statements about the relationship of DF and health, and the second section (Section B) included statements on the knowledge of DF content in some foods. Each statement had three possible responses (True/False/Not sure). A pool of 46 statements that belonged to these two sections was generated. Using this pool of statements, two reviews were carried out by a panel of three dieticians to select the best in terms of clarity of the questions, accuracy of the knowledge measured, and interpretability. This process reduced the number of statements to 20. After item analysis, the total number of statements in the final questionnaire was 18 (i.e. 9 in each section). Demographic questions on gender and age were included at the end of the questionnaire. The responses to the questionnaire took 3 to 5 minutes.

The questionnaire was self-administered in groups at the end of the lectures under the supervision of the authors. Some students completed the questionnaire on two separate occasions that were two weeks apart. The period of two weeks was considered long enough for participants to have forgotten their responses but not long enough for a real change to occur in their knowledge on nutrition (15-17). Participants were not informed of the second administration of the questionnaire on the first occasion.

The responses in the first administration were used in assessing construct validity and internal consistency reliability. Two sets of responses (i.e. the first and the second administration) were used in measuring test-retest reliability.

### Item analysis

Item analysis involves statistical analysis of the results of a test administration to identify which items can be retained and which need to be discarded (18). The results were analyzed for difficulty with and discrimination of the items, and only those items meeting the analysis criteria were retained.

### Difficulty with item

The item difficulty index indicates the percentage of respondents who answer an item correctly. Since difficulty refers to the percentage of getting the item right, the smaller the percentage figure, the more difficult the item is (18). According to Kline (19), items are not useful if they are answered correctly by more than 80% or fewer than 20% of the respondents. Items, therefore, were rejected if over 80% or under 20% of the respondents answered them correctly.

### Item discrimination

Item discrimination is the ability of an individual item to discriminate between those who do well on the measure and those who do not (16). An item-to-total score correlation of 0.20 has been given as the cutoff point below which items should be discarded (16,19,20), and, therefore, only the items that met the correlation of 0.20 or higher were retained.

### Final knowledge test

Those items that met the item analysis criteria formed the final knowledge questionnaire. Construct validity, internal consistency reliability, and test-retest reliability of the final questionnaire were assessed.

### Construct validity

Validity is the extent to which a test measures what it is intended to measure (8). One of the easiest ways to assess construct validity is to give the measure to two groups, one of which is known to have higher knowledge on nutrition than the other group (16).

### Internal consistency reliability

The tendency towards consistency found in repeated measurements of the same phenomenon is referred to as reliability (21). Internal consistency refers to the extent to which all of the items in a scale measure the different aspects of the same attribute (22). Cronbach's alpha is often used in assessing the reliability of tests for knowledge on nutrition, with questions that have more than two possible responses (8). Cronbach's alpha ranges from r=0 to 1, with r=0.7 or greater considered as sufficiently reliable (18).

### Test-retest reliability

Test-retest reliability involves administering the same measure to the same group of test-takers under the same conditions on two different occasions and correlating the scores (23). The reliability coefficient is simply the correlation (usually a Pearson's correlation) between the scores on the first and the second testing (17). The value for a Pearson's coefficient can fall between 0.00 (no correlation) and 1.00 (perfect correlation).

### Data collection and analysis

The raw data from responses of each participant were coded numerically. The responses were converted to 1 and 0 for correct and incorrect answers respectively (‘not sure’ responses were also coded as incorrect). Data were entered and analyzed using the Statistical Package for Social Sciences (version 17.0) (SPSS Inc., Chicago, IL, USA). A number of statistical tests were performed to assess the validity and reliability of the questionnaire. Student's *t*-test was employed to determine significant differences between the group means (p<0.001). Chi-square test was used in establishing whether the distribution of age and gender differed significantly between the two groups (p<0.05). The effect of the differences in age and gender on the results was checked by an analysis of covariance (ANCOVA), with significance level set at p<0.05.

**Table 1.** Gender and age of the study population (n=360)

Gender and age	Students who took nutrition courses (n=174)		Students who did not take nutrition courses (n=186)	
	n	%	n	%
Gender				
Male	5	2.9	61	32.8
Female	169	97.1	125	67.2
Age (years)				
18-<21	51	29.3	37	19.9
21-<25	123	70.7	140	75.3
25-32	0	0	9	4.8

## RESULTS

Compliance was high (90%) for the first test, with almost all students completing the questionnaire. Three hundred and sixty students completed the questionnaire at least once (174 students who took nutrition courses and 186 students who did not). Of them, 202 students (88 students who took nutrition courses and 114 students who did not) completed the questionnaire twice. There was a significant gender difference between the two groups, with 97.1% of the students who took nutrition courses and 67.2% of the students who did not take nutrition courses being female (p=0.000) (Table 1). This was due to the fact that majority of the students who took nutrition courses came from the departments that were dominated by females (such as nutrition and dietetics and nursing and health services). Majority of the students in both groups (70.7% of the students who took nutrition courses and 75.3% of the other group) were aged between 21 and 24 years. Difference in age between the two groups was significant (p=0.003).

### Construct validity

Table 2 shows that the students who studied nutrition scored consistently higher than the students in the other group on both sections of the questionnaire (p<0.001). The students who studied nutrition had an average score (mean±SD) of 12.7±7.4, and the other group had an average score (mean±SD) of 7.4±4.5 out of a maximum score of 18. The scores ranged from 2 to 18 in the group of students who studied nutrition and 0 to 16 in the other group. The students who studied nutrition answered more questions correctly in both Section A (6.5±1.8) and Section B (6.1±1.9) compared to the other group (3.5±2.4 and 4.0±2.5 for Section A and B respectively).

Given the different apparent gender balance of the two groups, gender was controlled for in an analysis of covariance but this did not have a significant effect on the results. When the same analysis was carried out for age, it was found that the difference in age did not affect the results significantly. The questionnaire, therefore, met the criterion for construct validity.

### Internal consistency reliability

Cronbach's alpha values in the two groups were different (Table 3). The values (individual sections and as cumulative) for the students who did not take nutrition courses were higher than for the other group. When all students were considered, the alpha values for both Section A and B were 0.83. The alpha value for the overall questionnaire was 0.90.

**Table 2.** Correct scores of the dietary fibre knowledge questionnaire expressed in mean, standard deviation, and range (n=360)

Knowledge section (Max score)	Students who took nutrition courses (n=174)				Students who did not take nutrition courses (n=186)				Difference between group means	
	Min	Max	Mean	SD	Min	Max	Mean	SD	Mean difference	p value
Part A: Dietary fibre and health (9)	1	9	6.5	1.8	0	9	3.5	2.4	3.0	0.000
Part B: Dietary fibre and foods (9)	0	9	6.1	1.9	0	9	4.0	2.5	2.1	0.000
Total (18)	2	18	12.7	7.4	0	16	7.4	4.5	5.3	0.000

Max=Maximum; Min=Minimum; SD=Standard deviation

**Table 3.** Internal and test-retest reliability

Knowledge section (Max score)	Internal reliability (Cronbach's α)			Test-retest reliability (Pearson's r)		
	Students who took nutrition courses (n=174)	Students who did not take nutrition courses (n=186)	Overall (n=360)	Students who took nutrition courses (n=88)	Students who did not take nutrition courses (n=114)	Overall (n=202)
Part A: Dietary fibre and health (9)	0.57	0.83	0.83	0.76*	0.84	0.88*
Part B: Dietary fibre and foods (9)	0.68	0.84	0.83	0.69*	0.84*	0.84*
Total (18)	0.75	0.90	0.90	0.80*	0.87*	0.90*

*p<0.001

**Table 4.** The responses with correct answers **(bold)** given to questions on dietary fibre-related knowledge (n=60)

Item number	Responses*		
	True (%)	False (%)	Not sure (%)
Part A: Dietary fibre and health			
1 Dietary fibre is a carbohydrate found in foods	**50.6**	21.7	27.8
2 Dietary fibre has high energy content	19.4	**54.2**	26.4
3 Regular consumption of dietary fibre may prevent colon cancer	**66.4**	3.3	30.3
4 Dietary fibre can help maintain our body-weight	**67.2**	9.2	23.6
5 Foods that contain dietary fibre should not be consumed by people who have diabetes	11.4	**54.4**	34.2
6 Regular consumption of dietary fibre may reduce blood cholesterol levels	**53.1**	8.9	38.1
7 When the consumption of dietary fibre increases, bowel movements will slow down	11.4	**66.9**	21.7
8 It is not necessary to consume foods that contain dietary fibre on a daily basis	22.8	**47.5**	29.7
9 Excessive consumption of dietary fibre may interfere with vitamin and mineral absorption	**33.6**	25.3	41.1
Part B: Dietary fibre and foods			
1 White bread has the highest dietary fibre content compared to other types of breads	11.9	**67.5**	20.6
2 Raw vegetables have a higher dietary fibre content compared to cooked ones	**69.7**	8.6	21.7
3 100 g of fruit and 100 g of fruit juice both contain the same amounts of dietary fibre	2.2	**78.1**	19.7
4 Milk and milk products do not contain dietary fibre	**42.2**	14.4	43.3
5 Legumes (such as beans, chickpeas, etc.) have the highest dietary fibre content	**44.2**	19.2	36.7
6 Dietary fibre intake can be increased by consuming more foods in the meat group (meat, poultry, and fish)	21.9	**36.4**	41.7
7 Nuts, such as hazelnuts and almonds, are rich in dietary fibre	**40.8**	17.5	41.7
8 Fruits with skin contain more dietary fibre than the ones without skin	**54.4**	7.8	37.8
9 Fast food consumption, in general, contributes to our dietary fibre intake	7.8	**71.4**	20.8

*Correct answers for each item given in **bold**

### Test-retest reliability

Pearson's correlation coefficient was calculated on the scores of the participants (88 students who studied nutrition and 114 students who did not study nutrition) who completed the questionnaire twice. The correlation coefficients varied across the sections and the two student groups (ranging from 0.69 to 0.87). The overall reliability was high (r=0.90, p<0.001) (Table 3).

### Responses

The most correctly-answered questions (66.4 to 67.2%) in Section A (DF and health) were about the effects of DF on health [i.e. prevention of colon cancer (A3), helping maintain body-weight (A4) and bowel movements (A7)] (Table 4). Four questions [(i.e. DF is a carbohydrate in foods (A1), DF has high energy content (A2), the intake of DF by people with diabetes (A5), and the effect of DF on blood cholesterol levels (A6)] were answered correctly by approximately half of the participants (range 50.6-54.4%). Item A8 was about the need for consuming DF on a daily basis and could only be answered by 47.5% of the participants. Item A9 enquired about the effect of the consumption of DF on vitamin and mineral absorption, and it was answered correctly by fewer participants than any other questions (33.6%).

The questions answered correctly most often in Section B (DF and foods) were about the DF content of the breads (B1), raw vegetables compared to cooked ones (B2), comparison of the DF content of fresh fruit and fruit juice (B3), and DF content of fast foods (B9) (range 67.5-78.1%). Only 54.4% of the participants answered question B8 (fruits with or without skins as a source of DF) correctly. The rest of the questions, which were about the food sources of fibre (B4, B5, B6, and B7), could be answered by 36.4 to 44.2% of the participants.

## DISCUSSION

Significant differences between the scores of the students who had taken a nutrition course for 14 weeks and the ones who had not taken this course indicated that the questionnaire had satisfactory construct validity, even when taking into account the skewed gender and age characteristics of the two groups. The students who had taken the nutrition course scored higher on both sections of the questionnaire. These students gave more correct answers for each statement (ranging from 51 to 72%) (data not shown). Majority of the “Not sure” responses were given by the group who had not taken the course (ranging from 61 to 90%) (data not shown). Therefore, it could be concluded that the nutrition course improved the knowledge level of the students who had taken the course.

The overall test-retest reliability value (0.90) was high, and this suggested that the questionnaire measured the knowledge of DF consistently over time. This value was slightly higher than in the studies that employed a general nutrition knowledge questionnaire in Australia (0.87) and Turkey (0.86) and lower than the value obtained for the same questionnaire in the UK (0.98) (15,24,25).

Nutrition scholars prefer internal reliability values of 0.70 or greater as an indication that a test is sufficiently reliable for measuring knowledge and knowledge structures (26). Cronbach's alpha values ranging from 0.5 to 0.8 (mostly between 0.6 and 0.7) were reported for studies that measured knowledge on nutrition (8,27,28). The internal reliability value of the questionnaire (0.90) was higher than these values and, therefore, the questionnaire could be considered a reliable tool to measure the knowledge on DF. This value is very similar to the values obtained from validation studies of a general nutrition knowledge questionnaire in Australia (25) and Turkey (24) (0.92 and 0.89, respectively) and lower than the UK study (0.97) (15).

Searching for studies that assessed knowledge on DF in Turkey produced only one result. However, a modified questionnaire was used in that study (13), and no statistical data on reliability and validity were presented. Therefore, it was not possible to compare the current results with those of that study.

One implication of this research is that a high number of students were unaware of the dietary recommendation about DF (item A5), and many students had a poor knowledge of the food sources of DF (item B4, B5, B6, and B7). It is strongly believed that these students were not able to make healthful food choices and meet the daily recommendation for DF due to their lack of knowledge. Without sufficient knowledge, individuals are not able to translate knowledge into the adoption of healthier food habits (29). While there are no data to compare our findings, studies have shown that university students can increase their knowledge on nutrition by taking relevant courses (30-32). However, further studies are necessary to verify this possible relationship between the knowledge and consumption of DF. This could be achieved by asking the participants to keep 3-day food diaries in addition to the employment of the DF questionnaire.

### Limitations

There are limitations in this research, and the main one concerns the sample selection. The sample was based on convenience. Due to the nature of the group that the sample was selected from (i.e. students studying dietetics and nursing), majority of the students were female. However, gender (as a potential confounding variable) was controlled for and did not have any significant effect on the results.

It is generally agreed that educated people demonstrate significantly better knowledge on nutrition (22,33). It is tempting to suggest that if this questionnaire was used in exploring the knowledge level of the general public on DF, more questions would be incorrectly answered by the participants.

### Conclusions

The questionnaire assessed in this study proved to be a valid and reliable tool to measure the knowledge on DF. Since it is easy to understand and it can be completed by the participants in a short time, it can be used in determining the knowledge level of other sub-populations or the general public more readily and consistently. Implementing the questionnaire would probably not increase knowledge on DF in the Turkish community but it could be used in identifying areas where people are the most deficient. This information could be used in developing public-health nutrition efforts to improve dietary habits and, in turn, influence nutrition-related disease risk.

## ACKNOWLEDGEMENTS

We acknowledge the contributions of the participants.
